# Supplementary material for: On-Treatment Changes in FIB-4 and 1-Year FIB-4 Values Help Identify Patients with Chronic Hepatitis B Receiving Entecavir Therapy Who Have the Lowest Risk of Hepatocellular Carcinoma
Source: Cancers (Basel). 2020 May 7;12(5):1177. doi: 10.3390/cancers12051177 (PMC7281667; doi:10.3390/cancers12051177)
Supplement: Supplementary file 1 [file cancers-12-01177-s001.pdf]

cancers

Article

On-Treatment Changes in FIB-4 and 1-Year FIB-4 Values Help Identify Patients with Chronic Hepatitis B Receiving Entecavir Therapy Who Have the Lowest Risk of Hepatocellular Carcinoma

Hung-Wei Wang, Hsueh-Chou Lai, Tsung-Hui Hu, Wen-Pang Su, Sheng-Nan Lu, Chia-Hsin Lin, Chao-Hung Hung, Po-Heng Chuang, Jing-Houng Wang, Mei-Hsuan Lee, Chien-Hung Chen and Cheng-Yuan Peng

Supplementary Materials:

Table S1. The values of noninvasive fibrosis indices in patients with CHB.

Noninvasive fibrosis index (Median ± IQR)	All patients (*n* = 1325)	Noncirrhotic (*n* = 844)	Cirrhotic (*n* = 481)
APRI	1.22 ± 2.1	1.22 ± 2.47	1.22 ± 1.63
APRI 12M	0.42 ± 0.37	0.34 ± 0.20	0.69 ± 0.57
FIB-4	2.45 ± 2.69	1.98 ± 2.03	3.22 ± 3.57
FIB-4 12M	1.64 ± 1.56	1.28 ± 0.98	2.61 ± 2.24

Abbreviations: APRI—AST/PLT ratio index; CHB—chronic hepatitis B; FIB-4—fibrosis index based on four factors; IQR—interquartile range; M—month; PLT—platelet.

Table S2. The sensitivity, specificity, PPV, NPV and accuracy according to the optimal cutoff values of APRI and FIB-4 indices by Youden index in predicting HCC risk.

Noninvasive fibrosis indices	Time point (month)	Optimal cutoff value	Sensitivity (%)	Specificity (%)	PPV (%)	NPV (%)	Accuracy (%)
**All patients (*n* = 1325)**							
APRI	0	0.90	76.2	36.6	9.38	94.7	39.8
	12	0.53	87.6	69.2	19.7	98.5	70.6
FIB-4	0	2.53	78.1	54.5	12.9	96.7	56.4
	12	2.56	81.0	78.9	24.8	98.0	79.0
**Noncirrhotic patients (*n* = 844)**							
APRI	0	0.91	75.0	36.9	1.69	99.0	37.4
	12	0.40	91.7	63.7	3.51	99.8	64.1
FIB-4	0	2.53	66.7	62.0	2.47	99.2	62.1
	12	1.58	91.7	65.0	3.64	99.8	65.4
**Cirrhotic patients (*n* = 481)**							
APRI	0	1.25	62.4	54.1	24.6	85.7	55.7
	12	0.80	72.0	65.5	33.3	90.7	66.7

FIB-4	0	3.90	59.1	61.9	27.1	86.3	61.3
	12	2.88	77.4	63.4	33.6	92.1	66.1

Abbreviations: APRI—AST/PLT ratio index; CI—confidence interval; FIB-4—fibrosis index based on four factors; HCC—hepatocellular carcinoma; PPV—positive predictive value; NPV—negative predictive value.

Table S3. Comparison of APRI and FIB-4 at the different time points to predict HCC risk by AUROC in CHB patients with or without cirrhosis.

**Noninvasive fibrosis indices**	**Time point (month)**	**AUROC (95% CI)**	**Pair-wise comparison of AUROC (*p* value)**			
**All patients**						
			APRI 0 month	APRI 12 months	FIB-4 0 month	FIB-4 12 months
APRI	0	0.539 (0.488–0.589)				
	12	0.860 (0.827–0.893)	< 0.0001			
FIB-4	0	0.694 (0.645–0.744)	< 0.0001	< 0.0001		
	12	0.872 (0.841–0.902)	< 0.0001	0.1930	< 0.0001	
**Noncirrhotic patients**						
			APRI 0 month	APRI 12 months	FIB-4 0 month	FIB-4 12 months
APRI	0	0.483 (0.333–0.632)				
	12	0.848 (0.756–0.940)	< 0.0001			
FIB-4	0	0.646 (0.499–0.794)	0.0001	0.0006		
	12	0.857 (0.749–0.964)	< 0.0001	0.7357	< 0.0001	
**Cirrhotic patients**						
			APRI 0 month	APRI 12 months	FIB-4 0 month	FIB-4 12 months
APRI	0	0.564 (0.502–0.625)				
	12	0.735 (0.680–0.790)	< 0.0001			
FIB-4	0	0.610 (0.547–0.673)	0.0087	< 0.0001		
	12	0.758 (0.707–0.810)	< 0.0001	0.1070	< 0.0001	

Abbreviations: APRI—AST/PLT ratio index; AUROC—area under the receiver operating characteristic; CHB—chronic hepatitis B; CI—confidence interval; FIB-4—fibrosis index based on four factors; HCC—hepatocellular carcinoma.

Table S4. Comparison of △APRI (12M-0M) and △FIB-4 (12M-0M) to predict HCC risk by AUROC in CHB patients with or without cirrhosis.

**Noninvasive fibrosis indices**	**AUROC (95% CI)**	**Pair-wise comparison of AUROC (*p* value)**	
**All patients**			
		△APRI (12M-0M)	△FIB-4 (12M-0M)
△APRI (12M-0M)	0.672 (0.613–0.732)		
△FIB-4 (12M-0M)	0.699 (0.633–0.765)	0.1096	
**Noncirrhotic patients**			
		△APRI (12M-0M)	△FIB-4 (12M-0M)
△APRI (12M-0M)	0.686 (0.494–0.877)		
△FIB-4 (12M-0M)	0.780 (0.571–0.989)	0.1264	
**Cirrhotic patients**			
		△APRI (12M-0M)	△FIB-4 (12M-0M)
△APRI (12M-0M)	0.605 (0.536–0.674)		
△FIB-4 (12M-0M)	0.673 (0.603–0.743)	0.0001	

Abbreviations: APRI—AST/PLT ratio index; AUROC—area under the receiver operating characteristic; CHB—chronic hepatitis B; CI—confidence interval; FIB-4—fibrosis index based on four factors; HCC—hepatocellular carcinoma; M—month.

Table S5. Multivariate Cox regression analysis of risk factors (with or without cirrhosis factor) associated with HCC in patients with CHB.

**Risk factors**	**Multivariate**	
	**Hazard ratio (95% CI)**	***p* value**
**All patients (*n* = 1325)**		
**Risk factor "cirrhosis" not included in multivariate Cox regression analysis**		
Diabetes mellitus, yes vs no	1.726 (1.076–2.770)	0.0235
AFP 12M, ng/mL	1.005 (1.003–1.008)	< 0.0001
FIB-4 12M, ≥ 2.56 vs < 2.56	9.198 (5.610–15.08)	< 0.0001
△FIB-4 (12M-0M), ≥ 0 vs < 0	2.353 (1.585–3.495)	< 0.0001
**Risk factor "cirrhosis" included in multivariate Cox regression analysis**		
Cirrhosis, yes vs no	5.174 (2.715–9.860)	< 0.0001
Diabetes mellitus, yes vs no	1.550 (0.965–2.489)	0.0700
AFP 12M, ng/mL	1.006 (1.003–1.008)	< 0.0001
FIB-4 12M, ≥ 2.56 vs < 2.56	5.107 (3.054–8.539)	< 0.0001
△FIB-4 (12M-0M), ≥ 0 vs < 0	2.387 (1.610–3.538)	< 0.0001
**Noncirrhotic patients (*n* = 844)**		
FIB-4 12M, ≥ 1.58 vs < 1.58	12.10 (1.531–95.60)	0.0181
△FIB-4 (12M-0M), ≥ 0 vs < 0	7.013 (1.874–26.24)	0.0038
**Cirrhotic patients (*n* = 481)**		
Sex, male vs female	1.758 (1.082–2.856)	0.0226
Diabetes mellitus, yes	1.665 (1.006–2.756)	0.0472
AFP 12M, ng/mL	1.008 (1.005–1.012)	< 0.0001
FIB-4 12M, ≥ 2.88 vs < 2.88	4.821 (2.908–7.992)	< 0.0001
△FIB-4 (12M-0M), ≥ 0 vs < 0	1.981 (1.301–3.016)	0.0014

Abbreviations: AFP—alpha–fetoprotein; CHB—chronic hepatitis B; CI—confidence interval; FIB-4—fibrosis index based on four factors; HCC—hepatocellular carcinoma; M—month.

Table S6. Univariate Cox regression analysis of risk factors associated with HCC in patients with noncirrhotic CHB.

Variables<br>Median ± IQR or *n* (%)	Noncirrhotic CHB		
	Hazard ratio	95% CI	*p* value
Age (year)	1.068	1.017–1.121	0.0087
Sex, male vs female	1.089	0.294–4.024	0.8987
HBeAg, positive vs negative	0.260	0.057–1.187	0.0820
Diabetes mellitus, yes vs no	2.061	0.451–9.417	0.3509
Albumin, g/dL	0.608	0.214–1.725	0.3497
AST, U/L	0.999	0.996–1.002	0.3907
ALT, U/L	0.997	0.992–1.001	0.1436
Total bilirubin, mg/dL	0.603	0.238–1.527	0.2861
INR	1.964	0.291–13.25	0.4882
Platelet, ×10^3/μL	0.985	0.973–0.998	0.0216
AFP, ng/mL	0.998	0.989–1.007	0.6509
AFP 12M, ng/mL	1.002	0.982–1.022	0.8555
HBV DNA, log IU/mL	0.901	0.629–1.291	0.5698
VR 12M, yes vs no	2.255	0.291–17.48	0.4363
APRI, ≥ 0.91 vs < 0.91	1.937	0.524–7.165	0.3216
APRI 12M, ≥ 0.40 vs < 0.40	18.46	2.383–142.9	0.0053
△APRI (12M-0M), ≥ 0 vs < 0	5.243	1.418–19.39	0.0130
FIB-4, ≥ 2.53 vs < 2.53	3.291	0.991–10.93	0.0518
FIB-4 12M, ≥ 1.58 vs < 1.58	18.57	2.396–143.8	0.0052
△FIB-4 (12M-0M), ≥ 0 vs < 0	11.03	2.985–40.75	0.0003

Abbreviations: AFP—alpha-fetoprotein; ALT—alanine aminotransferase; AST—aspartate aminotransferase; APRI—AST/PLT ratio index; CHB—chronic hepatitis B; CI—confidence interval; FIB-4—fibrosis index based on four factors; HBeAg—hepatitis B e antigen; HBV—hepatitis B virus; HCC—hepatocellular carcinoma; INR—international normalized ratio; IQR—interquartile range; M—month; PLT—platelet; VR—virological response.

Table S7. Univariate Cox regression analysis of risk factors associated with HCC in patients with cirrhotic CHB.

Variables<br>Median ± IQR or *n* (%)	Cirrhotic CHB		
	Hazard ratio	95% CI	*p* value
Age (year)	1.036	1.017–1.055	0.0001
Sex, male vs female	1.108	0.692–1.775	0.6704
HBeAg, positive vs negative	1.374	0.878–2.152	0.1646
Diabetes mellitus, yes vs no	1.887	1.148–3.101	0.0122
Albumin, g/dL	0.441	0.311–0.625	< 0.0001
AST, U/L	0.999	0.998–1.001	0.4218
ALT, U/L	0.999	0.998–1.000	0.2819
Total bilirubin, mg/dL	0.991	0.901–1.091	0.8575
INR	1.345	0.665–2.722	0.4096
Platelet, ×10^3/μL	0.993	0.989–0.998	0.0033
AFP, ng/mL	1.000	0.997–1.002	0.8010
AFP 12M, ng/mL	1.010	1.007–1.014	< 0.0001
HBV DNA, log IU/mL	0.943	0.813–1.094	0.4383

VR 12M, yes vs no	0.840	0.367–1.924	0.6798
APRI, ≥ 1.25 vs < 1.25	1.785	1.178–2.705	0.0063
APRI 12M, ≥ 0.80 vs < 0.80	4.250	2.695–6.700	< 0.0001
△APRI (12M-0M), ≥ 0 vs < 0	2.236	1.446–3.455	0.0003
FIB-4, ≥ 3.90 vs < 3.90	2.187	1.446–3.308	0.0002
FIB-4 12M, ≥ 2.88 vs < 2.88	4.930	3.028–8.026	< 0.0001
△FIB-4 (12M-0M), ≥ 0 vs < 0	2.700	1.794–4.063	< 0.0001

Abbreviations: AFP—alpha-fetoprotein; ALT—alanine aminotransferase; AST—aspartate aminotransferase; APRI—AST/PLT ratio index; CHB—chronic hepatitis B; CI—confidence interval; FIB-4—fibrosis index based on four factors; HBeAg—hepatitis B e antigen; HBV—hepatitis B virus; HCC—hepatocellular carcinoma; INR—international normalized ratio; IQR—interquartile range; M—month; PLT—platelet; VR—virological response.

Table S8. Characteristics of patients with noncirrhotic CHB stratified by △FIB-4 (*n* = 844).

**Variables Median ± IQR or *n* (%)**	**△FIB-4 <0 *n* = 674**	**△FIB-4 ≥0 *n* = 170**	***p* value**
**Baseline**			
Age (year)	47 ± 17	50 ± 15	0.013
Sex (male)	481 (71.4)	123 (72.4)	0.799
HBeAg-positive- status (yes)	298 (44.2)	57 (33.5)	0.012
Diabetes mellitus (yes)	69 (10.2)	14 (8.2)	0.433
HCC	3 (0.4)	9 (5.3)	< 0.001
Albumin, g/dL	4.2 ± 0.7	4.3 ± 0.6	0.038
AST, U/L	115 ± 254	48 ± 24	< 0.001
ALT, U/L	190 ± 476	78 ± 60	< 0.001
Total bilirubin, mg/dL	1.1 ± 1.1	0.8 ± 0.5	< 0.001
INR	1.1 ± 0.2	1.0 ± 0.1	< 0.001
Platelet, $\times 10^3/\mu L$	182 ± 70	189 ± 75	0.022
AFP, ng/mL	5.9 ± 13.0	3.8 ± 3.5	< 0.001
HBV DNA, log IU/mL	6.4 ± 2.4	5.5 ± 2.6	< 0.001
FIB-4	2.32 ± 2.32	1.38 ± 1.05	< 0.001
**One-year treatment**			
AST 12M, U/L	24 ± 9	27 ± 9	< 0.001
ALT 12M, U/L	24 ± 14	25 ± 19	0.052
Platelet 12M, $\times 10^3/\mu L$	190 ± 65	169 ± 68	< 0.001
AFP 12M, ng/mL	3.0 ± 1.7	3.1 ± 1.8	0.625
VR 12M (yes)	569 (84.4)	145 (85.3)	0.778
FIB-4 12M	1.20 ± 0.92	1.62 ± 1.23	< 0.001

Abbreviations: AFP—alpha-fetoprotein; ALT—alanine aminotransferase; AST—aspartate aminotransferase; CHB—chronic hepatitis B; DNA—deoxyribonucleic acid; FIB-4—fibrosis index based on four factors; HBeAg—hepatitis B e antigen; HBV—hepatitis B virus; HCC—hepatocellular carcinoma; INR—international normalized ratio; IQR—interquartile range; M—month; VR—virological response.

Table S9. Characteristics of patients with cirrhotic CHB stratified by △FIB-4 (*n* = 481).

**Variables Median ± IQR or *n* (%)**	**△FIB-4 <0 *n* = 328**	**△FIB-4 ≥0 *n* = 153**	***p* value**
**Baseline**			
Age (year)	53 ± 15	54 ± 15	0.840
Sex (male)	246 (75)	113 (73.9)	0.788
HBeAg-positive- status (yes)	69 (21)	51 (33.3)	0.004
Diabetes mellitus (yes)	51 (15.5)	24 (15.7)	0.969
HCC	42 (12.8)	51 (33.3)	< 0.001
Albumin, g/dL	4.0 ± 0.6	4.1 ± 0.5	0.428
AST, U/L	68 ± 88	44 ± 29	< 0.001
ALT, U/L	75 ± 116	50 ± 37	< 0.001
Total bilirubin, mg/dL	1.1 ± 0.8	1.0 ± 0.5	0.005
INR	1.1 ± 0.2	1.1 ± 0.1	0.028
Platelet, $\times 10^3/\mu L$	122 ± 67	139 ± 68	0.009
AFP, ng/mL	8.2 ± 13.6	6.0 ± 6.3	0.001
HBV DNA, log IU/mL	5.5 ± 2.2	5.2 ± 1.7	0.006
FIB-4	3.85 ± 3.97	2.59 ± 2.60	< 0.001
**One-year treatment**			
AST 12M, U/L	31 ± 12	37 ± 18	< 0.001
ALT 12M, U/L	32 ± 14	35 ± 18	< 0.001
Platelet 12M, $\times 10^3/\mu L$	129 ± 63	109 ± 68	< 0.001
AFP 12M, ng/mL	4.4 ± 3.2	4.7 ± 3.8	0.201
VR 12M (yes)	302 (92.1)	144 (94.1)	0.421
FIB-4 12M	2.47 ± 2.03	3.32 ± 3.80	< 0.001

Abbreviations: AFP—alpha-fetoprotein; ALT—alanine aminotransferase; AST—aspartate aminotransferase; CHB—chronic hepatitis B; DNA—deoxyribonucleic acid; FIB-4—fibrosis index based on four factors; HBeAg—hepatitis B e antigen; HBV—hepatitis B virus; HCC—hepatocellular carcinoma; INR—international normalized ratio; IQR—interquartile range; M—month; VR—virological response.

Table S10. Characteristics of all patients with CHB who developed HCC stratified by △FIB-4 (*n* = 105).

**Variables Median ± IQR or *n* (%)**	**△FIB-4 <0 *n* = 45**	**△FIB-4 ≥0 *n* = 60**	***p* value**
**Baseline**			
Age (year)	60 ± 15	56 ± 12	0.092
Sex (male)	39 (86.7)	40 (66.7)	0.019
HBeAg-positive- status (yes)	9 (20)	20 (33.3)	0.130
Diabetes mellitus (yes)	10 (22.2)	12 (20)	0.782
Cirrhosis status (yes)	42 (93.3)	51 (85)	0.184
Albumin, g/dL	3.9 ± 0.8	4.0 ± 0.8	0.688
AST, U/L	71 ± 103	53 ± 43	0.001
ALT, U/L	84 ± 132	59 ± 49	0.004
Total bilirubin, mg/dL	1.3 ± 0.8	0.9 ± 0.5	0.002
INR	1.1 ± 0.2	1.1 ± 0.2	0.836

Platelet, ×10³/μL	99 ± 65	127 ± 68	0.034
AFP, ng/mL	10.3 ± 31.0	7.3 ± 9.2	0.021
HBV DNA, log IU/mL	5.5 ± 2.2	5.5 ± 1.5	0.368
FIB-4	5.12 ± 4.89	3.10 ± 3.60	0.001
**One-year treatment**			
AST 12M, U/L	35 ± 15	39 ± 23	0.037
ALT 12M, U/L	31 ± 17	32 ± 12	0.209
Platelet 12M, ×10³/μL	98 ± 57	89 ± 51	0.041
AFP 12M, ng/mL	5.7 ± 5.5	5.5 ± 4.5	0.333
VR 12M (yes)	43 (95.6)	55 (91.7)	0.429
FIB-4 12M	3.91 ± 2.54	4.39 ± 4.17	0.039

Abbreviations: AFP—alpha-fetoprotein; ALT—alanine aminotransferase; AST—aspartate aminotransferase; CHB—chronic hepatitis B; DNA—deoxyribonucleic acid; FIB-4—fibrosis index based on four factors; HBeAg—hepatitis B e antigen; HBV—hepatitis B virus; HCC—hepatocellular carcinoma; INR—international normalized ratio; IQR—interquartile range; M—month; VR—virological response.

Table S11. Characteristics of patients with cirrhotic CHB who developed HCC stratified by △FIB-4 (n = 93).

Variables<br>Median ± IQR or *n* (%)	△FIB-4 <0<br>*n* = 42	△FIB-4 ≥0<br>*n* = 51	*p* value
**Baseline**			
Age (year)	59 ± 14	57 ± 13	0.116
Sex (male)	37 (88.1)	33 (64.7)	0.009
HBeAg-positive- status (yes)	9 (21.4)	18 (35.3)	0.143
Diabetes mellitus (yes)	10 (23.8)	10 (19.6)	0.624
Albumin, g/dL	3.9 ± 0.7	3.9 ± 0.8	0.957
AST, U/L	70 ± 96	51 ± 40	0.002
ALT, U/L	83 ± 129	52 ± 48	0.004
Total bilirubin, mg/dL	1.3 ± 0.7	0.9 ± 0.5	0.006
INR	1.1 ± 0.1	1.1 ± 0.2	0.677
Platelet, ×10³/μL	101 ± 64	123 ± 84	0.094
AFP, ng/mL	10.3 ± 29.4	7.9 ± 9.4	0.028
HBV DNA, log IU/mL	5.3 ± 2.2	5.4 ± 1.4	0.485
FIB-4	5.07 ± 3.91	3.19 ± 3.81	0.005
**One-year treatment**			
AST 12M, U/L	35 ± 15	40 ± 21	0.013
ALT 12M, U/L	31 ± 17	33 ± 12	0.105
Platelet 12M, ×10³/μL	97 ± 58	93 ± 57	0.038
AFP 12M, ng/mL	5.7 ± 5.6	6.1 ± 4.6	0.978
VR 12M (yes)	40 (95.2)	47 (92.2)	0.547
FIB-4 12M	3.94 ± 2.62	4.40 ± 4.57	0.038

Abbreviations: AFP—alpha-fetoprotein; ALT—alanine aminotransferase; AST—aspartate aminotransferase; CHB—chronic hepatitis B; DNA—deoxyribonucleic acid; FIB-4—fibrosis index based on four factors; HBeAg—hepatitis B e antigen; HBV—hepatitis B virus; HCC—hepatocellular carcinoma; INR—international normalized ratio; IQR—interquartile range; M—month; VR—virological response.

Table S12. Characteristics of patients with noncirrhotic CHB who developed HCC stratified by △FIB-4 (n = 12).

Variables<br>Mean ± SD or n (%)	△FIB-4 <0<br>n = 3	△FIB-4 ≥0<br>n = 9	p value
**Baseline**			
Age (year)	57.3 ± 9.3	56.6 ± 10.9	0.864
Sex (male)	2 (66.7)	7 (77.8)	1.000
HBeAg-positive- status (yes)	0 (0)	2 (22.2)	1.000
Diabetes mellitus (yes)	0 (0)	2 (22.2)	1.000
Albumin, g/dL	3.8 ± 0.8	4.0 ± 0.4	0.727
AST, U/L	326 ± 279	73 ± 47	0.282
ALT, U/L	193 ± 151	102 ± 81	0.209
Total bilirubin, mg/dL	1.5 ± 1.3	0.8 ± 0.3	0.727
INR	1.2 ± 0.4	1.1 ± 0.1	0.727
Platelet, ×10^3/μL	145 ± 86	151 ± 35	0.482
AFP, ng/mL	19.5 ± 25.2	20.7 ± 42.7	0.727
HBV DNA, log IU/mL	6.9 ± 1.0	5.8 ± 1.4	0.209
FIB-4	13.1 ± 10.5	2.85 ± 1.14	0.482
**One-year treatment**			
AST 12M, U/L	33 ± 15	37 ± 19	1.000
ALT 12M, U/L	34 ± 11	29 ± 8	0.482
Platelet 12M, ×10^3/μL	151 ± 87	105 ± 38	0.373
AFP 12M, ng/mL	8.4 ± 5.5	3.3 ± 1.4	0.036
VR 12M (yes)	3 (100)	8 (88.9)	1.000
FIB-4 12M	2.60 ± 1.67	4.27 ± 2.59	0.373

Abbreviations: AFP—alpha-fetoprotein; ALT—alanine aminotransferase; AST—aspartate aminotransferase; CHB—chronic hepatitis B; DNA—deoxyribonucleic acid; FIB-4—fibrosis index based on four factors; HBeAg—hepatitis B e antigen; HBV—hepatitis B virus; HCC—hepatocellular carcinoma; INR—international normalized ratio; IQR—interquartile range; M—month; VR—virological response.

Table S13. The AUROC, sensitivity, specificity, PPV, NPV and accuracy according to the cutoff values of APRI and FIB-4 indices for predicting HCC risk in cirrhotic patients with liver biopsy.

Noninvasive fibrosis indices	Time point (month)	Cutoff value	AUROC	Sensitivity (%)	Specificity (%)	PPV (%)	NPV (%)	Accuracy (%)
**Cirrhotic patients (n = 481)**		**Optimal**						
APRI	0	1.25	0.564	62.4	54.1	24.6	85.7	55.7
	12	0.80	0.735	72.0	65.5	33.3	90.7	66.7
FIB-4	0	3.90	0.610	59.1	61.9	27.1	86.3	61.3
	12	2.88	0.758	77.4	63.4	33.6	92.1	66.1
**Histology-verified F4 patients (n = 122)**		**Optimal**						
APRI	0	1.25	0.602	40.0	72.9	20.7	87.3	68.0
	12	0.80	0.918	86.7	74.1	37.1	96.9	76.0
FIB-4	0	3.90	0.608	40.0	77.6	24.0	88.0	72.0

	12	2.88	0.932	93.3	76.5	41.2	98.5	79.0
**Histology-verified F4 patients (*n* = 122)**		**Revised**						
APRI	0	1.62	0.602	40.0	82.4	28.6	88.6	76.0
	12	1.01	0.918	86.7	89.4	59.1	97.4	89.0
FIB-4	0	3.87	0.608	46.7	77.6	26.9	89.2	73.0
	12	2.58	0.932	100.0	74.1	40.5	100.0	NA

Abbreviations: APRI—AST/PLT ratio index; AUROC—area under the receiver operating characteristic; CI—confidence interval; FIB-4—fibrosis index based on four factors; HCC—hepatocellular carcinoma; PPV—positive predictive value; NPV—negative predictive value.

Table S14. Comparison of APRI and FIB-4 at the different time points to predict HCC risk by AUROC in cirrhotic patients with liver biopsy.

**Noninvasive fibrosis indices**	**Time point (month)**	**AUROC (95% CI)**	**Pair-wise comparison of AUROC (*p* value)**			
**Histology-verified F4 patients (*n* = 122)**						
			APRI 0 month	APRI 12 months	FIB-4 0 month	FIB-4 12 months
APRI	0	0.602 (0.449–0.756)				
	12	0.918 (0.846–0.991)	< 0.0001			
FIB-4	0	0.608 (0.447–0.769)	0.8831	0.0001		
	12	0.932 (0.879–0.985)	< 0.0001	0.6666	< 0.0001	

Abbreviations: APRI—AST/PLT ratio index; AUROC—area under the receiver operating characteristic; CI—confidence interval; FIB-4—fibrosis index based on four factors; HCC—hepatocellular carcinoma.

Table S15. Univariate and multivariate Cox regression analyses of risk factors associated with HCC in histology-verified cirrhotic patients (*n* = 122).

**Variables Median ± IQR or *n* (%)**	**Univariate**		**Multivariate**	
	**Hazard ratio (95% CI)**	***p* value**	**Hazard ratio (95% CI)**	***p* value**
Age (year)	1.049 (0.996–1.104)	0.072		
Sex, male vs female	0.708 (0.225–2.226)	0.555		
HBeAg, positive vs negative	2.688 (0.955–7.566)	0.061		
Diabetes mellitus, yes vs no	1.170 (0.330–4.152)	0.808		
Albumin, g/dL	0.278 (0.121–0.639)	0.003		
AST, U/L	0.999 (0.994–1.005)	0.853		
ALT, U/L	0.998 (0.992–1.005)	0.642		
Total bilirubin, mg/dL	1.218 (0.475–3.119)	0.681		
INR	31.35 (0.395–2485.6)	0.123		
Platelet, $\times 10^3/\mu L$	0.996 (0.985–1.007)	0.492		
AFP, ng/mL	1.004 (0.996–1.012)	0.326		
AFP 12M, ng/mL	1.135 (1.056–1.219)	0.001		

HBV DNA, log IU/mL	1.388 (0.966–1.994)	0.076
VR 12M, yes vs no	0.419 (0.094–1.859)	0.252
APRI, ≥ 1.62 vs < 1.62	2.864 (1.015–8.079)	0.047
APRI 12M, ≥ 1.01 vs < 1.01	34.73 (7.658–157.5)	< 0.001
△APRI (12M-0M), ≥ 0 vs < 0	3.984 (1.444–10.99)	0.008
FIB-4, ≥ 3.87 vs < 3.87	2.504 (0.905–6.927)	0.077
FIB-4 12M, ≥ 2.58 vs < 2.58	155.2 (1.546–15577)	0.032
△FIB-4 (12M-0M), ≥ 0 vs < 0	7.971 (2.248–28.26)	0.001

Abbreviations: AFP—alpha-fetoprotein; ALT—alanine aminotransferase; AST—aspartate aminotransferase; APRI—AST/PLT ratio index; CHB—chronic hepatitis B; CI—confidence interval; DNA—deoxyribonucleic acid; FIB-4—fibrosis index based on four factors; HBeAg—hepatitis B e antigen; HBV—hepatitis B virus; HCC—hepatocellular carcinoma; INR—international normalized ratio; IQR—interquartile range; M—month; PLT—platelet; VR—virological response.

Table S16. The AUROCs according to APRI and FIB-4 indices for predicting HCC risk in CHB patients receiving 3 years and 5 years of continuous ETV therapy.

Noninvasive fibrosis indices	Time point (month)	Cutoff value	Overall AUROC	ETV ≥ 3yr AUROC	ETV ≥ 5yr AUROC
**All patients**			*n* = 1325	*n* = 1053	*n* = 472
APRI	0	0.90	0.539	0.539	0.480
	12	0.53	0.860	0.868	0.839
FIB-4	0	2.53	0.694	0.700	0.670
	12	2.56	0.872	0.886	0.869
**Noncirrhotic patients**			*n* = 844	*n* = 679	*n* = 244
APRI	0	0.91	0.483	0.525	0.424
	12	0.40	0.848	0.924	0.988
FIB-4	0	2.53	0.646	0.705	0.881
	12	1.58	0.857	0.913	0.996
**Cirrhotic patients**			*n* = 481	*n* = 374	*n* = 228
APRI	0	1.25	0.564	0.559	0.492
	12	0.80	0.735	0.733	0.705
FIB-4	0	3.90	0.610	0.613	0.584
	12	2.88	0.758	0.772	0.762

Abbreviations: APRI—AST/PLT ratio index; AUROC—area under the receiver operating characteristic; ETV—entecavir; FIB-4—fibrosis index based on four factors; HCC—hepatocellular carcinoma.

Table S17. The sensitivity, specificity, PPV, NPV and accuracy according to the cutoff values of APRI and FIB-4 indices in predicting HCC risk in CHB patients receiving 3 years of continuous entecavir therapy (*n* = 1053).

Noninvasive fibrosis indices	Time point (month)	Cutoff value	Sensitivity (%)	Specificity (%)	PPV (%)	NPV (%)	Accuracy (%)
**All patients (*n* = 1053)**							
APRI	0	0.90	74.1	37.5	6.47	96.1	39.5

	12	0.53	89.7	69.0	14.4	99.1	70.2
FIB-4	0	2.53	79.3	55.5	9.41	97.9	56.8
	12	2.56	82.8	79.3	18.9	98.7	79.5
**Noncirrhotic patients (*n* = 679)**							
APRI	0	0.91	71.4	37.5	1.18	99.2	37.8
	12	0.40	100	65.3	2.92	100	NA
FIB-4	0	2.53	71.4	62.8	1.96	99.5	62.9
	12	1.58	100	64.9	2.88	100	NA
**Cirrhotic patients (*n* = 374)**							
APRI	0	1.25	60.8	54.8	17.5	89.8	55.6
	12	0.80	70.6	66.3	24.8	93.4	66.8
FIB-4	0	3.90	58.8	61.6	19.5	90.5	61.2
	12	2.88	78.4	63.5	25.3	94.9	65.5

Abbreviations: APRI—AST/PLT ratio index; CHB—chronic hepatitis B; FIB-4—fibrosis index based on four factors; HCC—hepatocellular carcinoma; PPV—positive predictive value; NPV—negative predictive value.

Table S18. The sensitivity, specificity, PPV, NPV and accuracy according to the cutoff values of APRI and FIB-4 indices in predicting HCC risk in CHB patients receiving 5 years of continuous entecavir therapy (*n* = 472).

Noninvasive fibrosis indices	Time point (month)	Cutoff value	Sensitivity (%)	Specificity (%)	PPV (%)	NPV (%)	Accuracy (%)
**All patients (*n* = 472)**							
APRI	0	0.90	60.0	40.9	3.23	96.9	41.5
	12	0.53	93.3	61.7	7.41	99.6	62.7
FIB-4	0	2.53	73.3	52.3	4.80	98.4	53.0
	12	2.56	80.0	74.2	9.23	99.1	74.4
**Noncirrhotic patients (*n* = 244)**							
APRI	0	0.91	100	42.4	0.71	100	NA
	12	0.40	100	63.0	1.10	100	NA
FIB-4	0	2.53	100	63.4	1.11	100	NA
	12	1.58	100	62.6	1.09	100	NA
**Cirrhotic patients (*n* = 228)**							
APRI	0	1.25	50.0	56.5	7.00	94.5	56.1
	12	0.80	64.3	66.4	11.1	96.6	66.2
FIB-4	0	3.90	57.1	62.6	9.09	95.7	62.3
	12	2.88	78.6	63.6	12.4	97.8	64.5

Abbreviations: APRI—AST/PLT ratio index; CHB—chronic hepatitis B; FIB-4—fibrosis index based on four factors; HCC—hepatocellular carcinoma; PPV—positive predictive value; NPV—negative predictive value.

Table S19. Comparison of PAGE-B, REACH-B, CU-HCC, and APA-B scores and FIB-4-based model by C-statistic and AUROCs to predict HCC risk at 3 and 5 years in patients with CHB receiving entecavir therapy.

Risk scores	AUROC (95% CI)	Pair-wise comparison of AUROC (p value)				
	**C-statistic**					
		**PAGE-B**	**REACH-B**	**CU-HCC**	**APA-B**	**FIB-4-based model[1]**
**PAGE-B**	0.7394 (0.6920–0.7868)					
**REACH-B**	0.6551 (0.6033–0.7069)	0.0004				
**CU-HCC**	0.7755 (0.7366–0.8145)	0.1795	0.0001			
**APA-B**	0.8825 (0.8532–0.9118)	< 0.0001	< 0.0001	< 0.0001		
**FIB-4-based model[1]**	0.8736 (0.8434–0.9038)	< 0.0001	< 0.0001	< 0.0001	0.4798	
	**3 years**					
		**PAGE-B**	**REACH-B**	**CU-HCC**	**APA-B**	**FIB-4-based model[1]**
**PAGE-B**	0.7415 (0.6741–0.8089)					
**REACH-B**	0.6640 (0.5950–0.7329)	0.0475				
**CU-HCC**	0.7771 (0.7210–0.8333)	0.3353	0.0092			
**APA-B**	0.8820 (0.8393–0.9247)	< 0.0001	< 0.0001	0.0001		
**FIB-4-based model[1]**	0.8359 (0.7858–0.8860)	0.0125	< 0.0001	0.0208	0.0206	
	**5 years**					
		**PAGE-B**	**REACH-B**	**CU-HCC**	**APA-B**	**FIB-4-based model[1]**
**PAGE-B**	0.7471 (0.6980–0.7962)					
**REACH-B**	0.6535 (0.5992–0.7078)	0.0003				
**CU-HCC**	0.7809 (0.7394–0.8225)	0.2191	0.0001			
**APA-B**	0.8775 (0.8450–0.9100)	< 0.0001	< 0.0001	< 0.0001		
**FIB-4-based model[1]**	0.8659 (0.8321–0.8997)	< 0.0001	< 0.0001	< 0.0001	0.3979	

Abbreviations: AUROC—area under the receiver operating characteristic; CHB—chronic hepatitis B; CI—confidence interval; FIB-4—fibrosis index based on four factors; HCC—hepatocellular carcinoma.

[1]FIB-4-based model is the combination of FIB-4 12M and △FIB-4 (12M-0M) for stratification of HCC risk.

Table S20. Cumulative incidence of HCC at 2 to 5 years of entecavir therapy in low-risk patients with CHB according to different prediction models.

Low-risk Group (percentage, case number of eligible patients)	Year 2 (%)	Year 3 (%)	Year 4 (%)	Year 5 (%)
**PAGE-B (<10) (15.8%, *n* = 209 of 1325)**	0.5	0.5	0.5	2.0
**REACH-B (≤8)[1] (9.2%, *n* = 78 of 844)**	0	0	0	0
**CU-HCC (<5) (35.4%, *n* = 469 of 1325)**	0.2	0.5	0.5	1.9
**APA-B (≤5) (75.9%, *n* = 1006 of 1325)**	0.5	0.9	1.4	2.6
**FIB-4-based model[2] (89.6%, *n* = 756 of 844)**	0.3	0.3	0.3	0.6

Abbreviations: CHB—chronic hepatitis B; FIB-4—fibrosis index based on four factors; HCC—hepatocellular carcinoma.

[1]Only patients without cirrhosis were analyzed for the REACH-B score.

[2]The low-risk group was noncirrhotic patients with FIB-4 12M < 1.58 and FIB-4 12M ≥ 1.58/△FIB-4 <0.

Table S21. Baseline and on-treatment characteristics of patients with CHB before multiple imputation.

Variables Median ± IQR or *n* (%)	Total *n* = 1325	HCC occurrence		*p* value
		Yes (*n* =105)	No (*n* = 1220)	
Age (year)	50 ± 17	58 ± 14	49 ± 17	< 0.0001
Sex (male)	963 (72.7)	79 (75.2)	884 (72.5)	0.5397
HBeAg-positive-status	475 (35.8)	29 (27.6)	446 (36.6)	0.0669
Missing (%)	0	0	0	
Cirrhosis status	481 (36.3)	93 (88.6)	388 (31.8)	< 0.0001
Missing (%)	0	0	0	
Diabetes mellitus (yes)	158 (11.9)	22 (21.0)	136 (11.1)	0.0029
Missing (%)	0	0	0	
Albumin, g/dL	4.1 ± 0.6	3.9 ± 0.8	4.2 ± 0.6	< 0.0001

Missing (%)	3.62	0.95	3.85	
AST, U/L	72 ± 115	60 ± 50	73 ± 123	0.0027
Missing (%)	0.15	0	0.16	
AST 12M, U/L	27 ± 12	37 ± 18	27 ± 11	< 0.0001
Missing (%)	0.15	0	0.16	
ALT, U/L	106 ± 207	64 ± 61	113 ± 220	< 0.0001
Missing (%)	0.15	0	0.16	
ALT 12M, U/L	27 ± 16	32 ± 13	26 ± 17	< 0.0001
Missing (%)	0.08	0	0.08	
Total bilirubin, mg/dL	1.0 ± 0.8	1.1 ± 0.7	1.0 ± 0.8	0.5566
Missing (%)	0.45	0	0.49	
INR	1.1 ± 0.1	1.1 ± 0.2	1.1 ± 0.1	0.0123
Missing (%)	3.25	2.86	3.28	
Platelet, ×10^3/μL	163 ± 76	120 ± 71	167 ± 74	< 0.0001
Missing (%)	0.30	0	0.33	
Platelet 12M, ×10^3/μL	163 ± 81	96 ± 52	169 ± 77	< 0.0001
Missing (%)	3.17	1.90	3.28	
Histology grading				
F 1/2/3/4/NA	57/84/30/122/1032	0/2/1/17/85	57/82/29/105/947	
A 0/1/2/3/NA	40/138/64/51/1032	3/11/2/4/85	37/127/62/47/947	
AFP, ng/mL	6.0 ± 10.2	8.3 ± 12.5	5.8 ± 10.0	< 0.0001
Missing (%)	0.75	0.95	0.74	
AFP 12M, ng/mL	3.4 ± 2.4	5.6 ± 4.7	3.3 ± 2.2	< 0.0001
Missing (%)	0.60	0	0.66	
HBV DNA, log IU/mL	5.96 ± 7.28	5.49 ± 6.46	6.04 ± 7.34	< 0.0001
Missing (%)	0	0	0	
VR 12M (yes)	1160 (87.5)	98 (93.3)	1062 (87)	0.0613
Missing (%)	0	0	0	
APRI	1.22 ± 2.13	1.43 ± 1.74	1.19 ± 2.16	0.1924
Missing (%)	0.45	0	0.49	
APRI 12M	0.43 ± 0.37	1.04 ± 0.68	0.41 ± 0.31	< 0.0001
Missing (%)	0.83	0	0.90	
FIB-4	2.45 ± 2.69	4.09 ± 4.05	2.37 ± 2.51	< 0.0001
Missing (%)	1.28	0	1.39	
FIB-4 12M	1.64 ± 1.57	4.09 ± 3.37	1.55 ± 1.39	< 0.0001
Missing (%)	0.83	0	0.90	
Treatment duration (year)	4.09 (2.94)	3.29 (2.32)	4.11 (3.00)	< 0.0001
Follow-up period (year)	4.09 (2.94)	3.29 (2.32)	4.11 (3.00)	< 0.0001

Abbreviations: AFP—alpha-fetoprotein; ALT—alanine aminotransferase; AST—aspartate aminotransferase; APRI—AST/PLT ratio index; CHB—chronic hepatitis B; DNA—deoxyribonucleic acid; FIB-4—fibrosis index based on four factors; HBeAg—hepatitis B e antigen; HBV—hepatitis B virus; HCC—hepatocellular carcinoma; INR—international normalized ratio; IQR—interquartile range; M—month; NA—not available; PLT—platelet; VR—virological response.

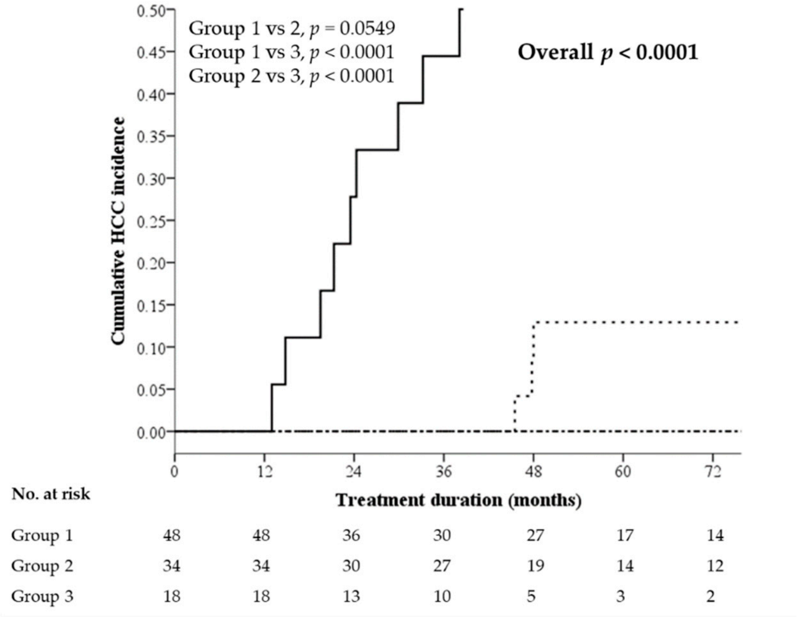

Figure S1. HCC risk stratification by a combination of FIB-4 12M and △FIB-4 in cirrhotic patients with liver biopsy: - · – Group 1, FIB-4 12M < 2.88 and △FIB-4 < 0; ···· Group 2 FIB-4 12M ≥ 2.88 and △FIB-4 < 0 or FIB-4 12M < 2.88 and △FIB-4 ≥ 0; —— Group 3 FIB-4 12M ≥ 2.88 and △FIB-4 ≥ 0.

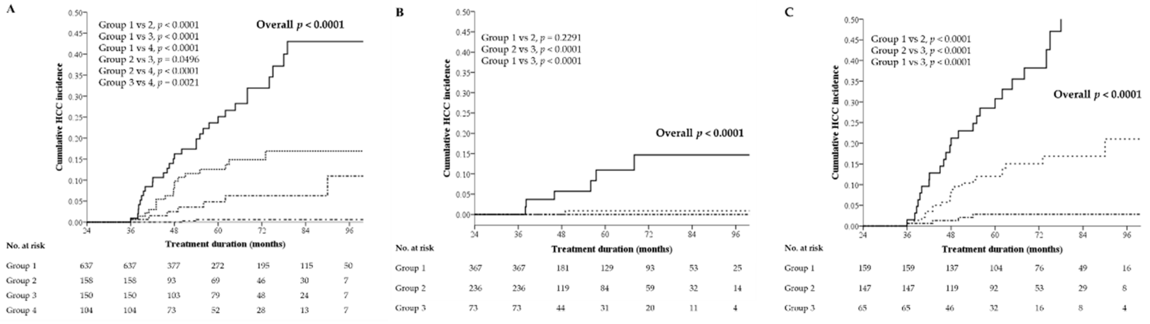

Figure S2. HCC risk stratification by a combination of FIB-4 12M and △FIB-4 in (A) all patients treated with entecavir ≥ 3 years: - —— Group 1, FIB-4 12M < 2.56 and △FIB-4 < 0; ▪▪▪▪ Group 2, FIB-4 12M < 2.56 and △FIB-4 ≥ 0; ···· Group 3, FIB-4 12M ≥ 2.56 and △FIB-4 < 0; —— Group 4, FIB-4 12M ≥ 2.56 and △FIB-4 ≥ 0; (B) noncirrhotic patients treated with entecavir ≥ 3 years: - · – Group 1, FIB-4 12M < 1.58 and △FIB-4 < 0; ···· Group 2, FIB-4 12M ≥ 1.58 and △FIB-4 < 0 or FIB-4 12M < 1.58 and △FIB-4 ≥ 0; —— Group 3, FIB-4 12M ≥ 1.58 and △FIB-4 ≥ 0 and (C) cirrhotic patients treated with entecavir ≥ 3 years: - · – Group 1, FIB-4 12M < 2.88 and △FIB-4 < 0; ···· Group 2 FIB-4 12M ≥ 2.88 and △FIB-4 < 0 or FIB-4 12M < 2.88 and △FIB-4 ≥ 0; —— Group 3 FIB-4 12M ≥ 2.88 and △FIB-4 ≥ 0.

Supplementary Methods. Details on multiple imputation.

Missing data assumed as missing at random were imputed with substituted values by using multiple imputation by chained equations [1]. The estimated value was extracted ten times from the distribution to form data sets. For each multiple imputation data set, we calculated the mean,

variance, minimum or maximum of the variables and combined the estimates by using Rubin's rules [2]. These substituted values in imputed data sets were limited within rational ranges. The imputed variables (percentage of missing data) were albumin (3.62%), AST (0.15%), ALT (0.15%), total bilirubin (0.45%), INR (3.25%), platelet (0.30%), AFP (0.75%), APRI (0.45%), FIB-4 (1.28%) at baseline; and AST 12M (0.15%), ALT 12M (0.08%), platelet 12M (3.17%), AFP 12M (0.60%), APRI 12M (0.83%), FIB-4 12M (0.83%) at one year of treatment (Table S19).

References

1. Little RJA, Rubin DB. Statistical analysis with missing data. Wiley. 2002.

2. Rubin DB, Schenker N. Multiple imputation in health-care databases: an overview and some applications. *Stat Med*. 1991, *10*, 585-598.
